# Control of epithelial homeostasis by apical polarity: it takes a network

**DOI:** 10.1042/BST20243002

**Published:** 2025-08-18

**Authors:** Sophie A. Lelièvre, Joséphine Briand

**Affiliations:** Relation Genes-Environment (REGEN) Unit, Department of Translational Research, Institut de Cancérologie de l’Ouest (ICO), Angers, Pays de la Loire, 49055, France

**Keywords:** apical junctional complex, higher order chromatin organization, primary prevention, breast cancer risk, gap junction, Connexin 43

## Abstract

The maintenance of cell functions in response to various stimuli is fulfilled by tightly controlled homeostatic processes. The basoapical structure of normal epithelia is increasingly recognized as the guardian of homeostasis. It has recently been demonstrated that apical polarity, depicted by lateroapical tight junctions, is controlled by gap junctions and sets the resting membrane potential, itself essential for homeostasis, in the breast luminal epithelium. In the breast, the disruption of apical polarity is recognized as a necessary step toward cancer onset, which calls for a better understanding of its consequences on the mechanisms of homeostasis all the way to the genome. Here, we extend the traditional apical junctional complex to include gap junctions and investigate its relation with epigenetically- driven and higher order chromatin organization. The disruption of apical polarity affects different types of molecular networks that remodel chromatin with a tendency toward openness or relaxation, a status typically associated with instability and cancer onset. Events known to foster the development of cancers, such as chronic inflammation, oxidative stress, stiffer microenvironment, and aging, are all triggering the disruption of apical polarity, which leads us to explore possibilities to re-establish full polarity. Focusing on gap junction intercellular communication mediated by Connexin 43 might be an interesting therapeutic option for retinoic acid derivatives. However, in light of the different degrees of apical polarity loss, we surmise that the resulting chromatin alterations might depend on the way apical polarity is disrupted initially, which suggests that therapeutic combinations targeted also toward these alterations might be required.

## Introduction

Apical polarity is the ultimate architectural differentiation feature of epithelia. It is essential to regulate homeostasis, an equilibrium necessary for normal functions, by enabling the adaptation of cells to microenvironmental changes along the life course. Loss of homeostasis normally occurs progressively during aging, that is characterized by chronic inflammation and oxidative stress [1], with the possibility of cell senescence, but it may also be associated with a drift in phenotype leading to cancer.

Apical polarity is illustrated by the lateroapical location of tight junctions (TJs) following the establishment of basal polarity represented by hemidesmosomes. It has been historically described as the regulator of paracellular permeability for the exchange of molecules between the apical and basal sides of the epithelium, or between the outside and the inside of the body. Recently, thanks to three-dimensional (3D) cell culture models of the breast epithelium, apical polarity-mediated homeostasis has been extended to the orientation of the mitotic spindle [2,3] and the setting of the resting membrane potential (RMP) [4]. The alteration of both features is associated with loss of homeostasis, since mitotic spindle misorientation may be the source of abnormal cell multilayering [2], and a change in the RMP level accompanies cancer development [4].

Apical polarity is maintained, directly or indirectly, via a structural network involving TJ, adherens junctions [5], the cytoskeleton [6], and gap junctions [2,7]. It is also controlled by the phosphoinositide 3-kinase (PI3K)-Akt pathway [2,8], the backbone of biochemical signaling networks involved in homeostasis [9]. PI3KT-Akt dysregulation participates in the development of different carcinomas, like breast cancers[10]. The loss of apical polarity is a characteristic of aging [11] and cancer [12], and we, along with others, have proposed that it is a necessary step in cancer onset [13,14]. Interestingly, although epithelial cells can divide within a normal polarized epithelium [15], it is only when apical polarity has been compromised in breast glandular structures produced in 3D cell culture that cells may enter the cell cycle in response to factors known to stimulate proliferation [2,3,16]. Moreover, in the absence of apical polarity in the tissue, cell proliferation leads to multilayering via random orientation of the mitotic spindle, which is an abnormal situation. Hence, under at-risk conditions (i.e. a tissue improperly polarized), we propose that re-establishing apical polarity may participate in cancer prevention.

Aging- and disease-related drift away from differentiation involves alterations in gene transcription regulation, notably through changes in chromatin [17]. Thus, deciphering the relation of apical polarity with chromatin should enhance our grasp of homeostasis.

Chromatin is made by the combination of DNA and histones, the chemical modifications of which form the epigenome involved in gene expression regulation. The epigenome interplays with a higher order of chromatin organization (HOCO) [18] proposed, early on, as the nuclear controller of cell homeostasis [19]. Progressive unraveling of HOCO dynamics has revealed that it is a complex combination of spatial epigenetic regions (made of similar euchromatin or heterochromatin marks) and chromatin loops. Differentiation is illustrated by changes in the strength of enhancer–promoter interactions (usually through chromatin looping) and of epigenetic regions, and by alterations in gene insulation by topologically associating domains (TAD) [20]. As a matter of fact, totipotent cells that present high phenotypic plasticity and thus low capacity to maintain their status through cell homeostasis are characterized by weak HOCO features, with a relaxed chromatin architecture [21].

Overall, epithelial homeostasis is supported by a strong cellular architecture, from cell membrane structures to HOCO. The latter locks in place regulatory nodes but with sufficient dynamicity in its components to ‘re-equilibrate’ cells when necessary. Yet, the labile characteristic of apical polarity in response to chemical and physical stressors [2,3,22,23] requires revisiting its interaction with HOCO. The considerable progress made in understanding HOCO’s relation to the epigenome and its involvement in homeostasis leads us in the first part to analyze how TJ-mediated apical polarity participates in cell homeostasis by influencing chromatin structure. Then, using the breast epithelium as an example, in a second part, we reflect on the possibilities to restore homeostasis following apical polarity loss.

## Part 1: the influence of apical polarity on chromatin

**Figure 1:**
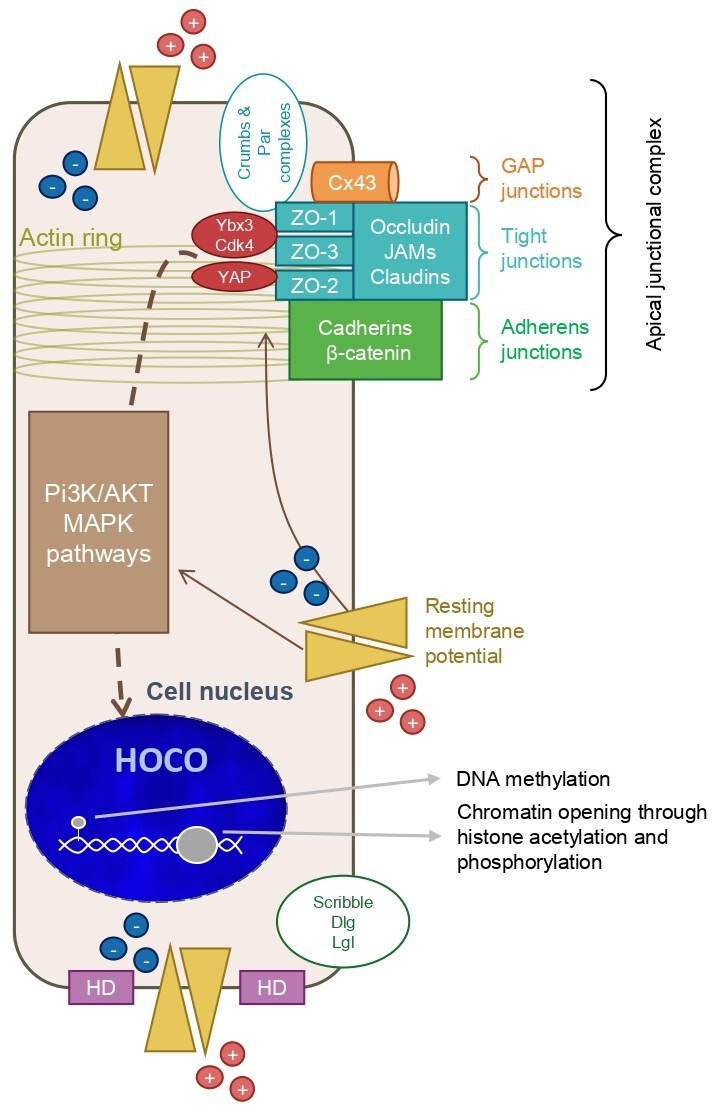
The apical junctional complex (AJC) interacts with many partners, networking all the way to the cell nucleus. The AJC is connected to the actin cytoskeleton, and typically formed by Crumbs and Par complexes, tight junctions (TJs), and adherens junctions, to which Cx43-mediated gap junctions may be added in the breast luminal epithelium. Apical polarity is formed upon the establishment of basolateral polarity depicted by cell–extracellular matrix junctions called hemidesmosomes (HD) and the Scribble protein cluster. Although several AJC proteins like ZO and β-catenin are constantly shuttling between the AJC and the cell nucleus, nonAJC molecules like transcription factors (TFs) Ybx3 interacting with CDK4 and YAP are usually trapped in the AJC when apical polarity is intact. Apical polarity also controls the level of resting membrane potential, as shown recently. Disruption of apical polarity leads to changes in chromatin, with local epigenetic modifications and HOCO, through the release of TFs, the activation of PI3K and MAPK pathways, and changes in the concentration of ions, like Ca^2+^, as a result of membrane voltage alterations that activate enzymes and TFs. CDK4, cyclin-dependent kinase 4; Cx43, Connexin 43; HOCO, higher order chromatin organization; JAMs, junctional adhesion molecules; MAPK, mitogen-activated protein kinase; Par, partitioning defective; Pi3K/AKT, phosphoinositide 3-kinase/Ak strain transforming; Ybx3, Y-box binding protein 3; YAP, yes-associated protein; ZO, zonula occludens.

The core of apical polarity is made of TJ located at the apex of epithelia, themselves connected to protein clusters (Crumbs and partitioning defective (Par) that contain regulatory factors of apical polarity), adherens junctions, and the cytoskeleton to form the apical junctional complex (AJC). Gap junctions that control the apical polarity may be considered part of the AJC in the breast because they are located lateroapically. There is also the Scribble cluster that is located basally. It keeps proteins of the AJC away from basolateral sites, although its exact role in the maintenance of apical polarity in mammals depends on tissue types. In the mammary gland, its disruption is associated with ductal hyperplasia [24] (Figure 1). Some of these elements directly act on the transcription of specific genes, notably the shuttling TJ proteins zonula occludens (ZO) and adherens junction protein β-catenin that, like ZO-2, interacts with Connexin 43 (Cx43), itself responsible for gap junctions in luminal breast epithelial cells [2,25]. The level of the RMP, an unavoidable regulator of homeostasis through ion channels and fluxes, is influenced by the presence of apical polarity [4]. There is currently no information on how bioelectrical signaling and gap junctions might work together to control homeostasis. One possibility that we have proposed previously might encompass the electrical coupling of ion channels located apically and involved in homeostasis, since gap junctions are lateroapical in the polarized breast epithelium [4]. Bioelectrical signaling reaches genes through enzyme-dependent cascades that control the post-translational modification of transcriptional regulators and activate signaling pathways. For instance, in cerebellar granule cells, membrane hyperpolarization inhibits calcineurin phosphatase, maintaining the transcriptional regulator ‘myocyte enhancer factor 2A’ in its active form and allowing it to play its transcriptional repressor role with a crucial effect in differentiation [26]. Interestingly, hyperpolarization activates the calcineurin pathway during myogenic differentiation [27], demonstrating the dual role of hyperpolarization on differentiation, depending on the cell type. In the next sections, we give specific examples of the impact of altered apical polarity on chromatin that contributes to loss of homeostasis.

### The nuclear impact of shuttling AJC proteins depends on the status of apical polarity

The sequestration of ZO-associated nucleic acid protein (DbpA), a transcription factor (TF) also called Y-box binding protein 3 (Ybx3), and its ligand cyclin-dependent kinase 4 (CDK4) at TJs in polarized (differentiated) cells is thought to inhibit G1/S phase transition [28]. In the absence of apical polarity, Ybx3 travels to the cell nucleus and binds responsive elements in the promoters of genes coding for proliferating cell nuclear antigen (PCNA) and cyclin D1 (an activator of CDK4), as shown with breast epithelial cells [29]. Cyclin D1 can associate with histone acetyltransferases p300 and P300/CBP-associated factor and histone deacetylases (HDAC) to regulate epigenetic changes at specific promoters [30]. The involvement of Ybx3 was also reported for the switch between polarized differentiation and proliferation in proximal tubular epithelial cells and intestinal goblet cells [31,32].

Another TF trapped at TJ is yes-associated protein (YAP), which associates with TAZ in Hippo signaling [33]. These TFs are activated through RHO family GTPase signaling and interact with other TFs, notably TEAD4, in the nucleus. YAP may repress or promote gene transcription. The interaction of YAP/TEAD4 with RAD21 triggers NuRD complex recruitment to act in a corepressive manner, as shown in ovarian cancer [34]. YAP recruits enhancer of zeste homolog 2 (EZH2), the catalytic subunit of polycomb repressive complex 2 implicated in H3K27 methylation, leading to the silencing of Jumonji domain-containing protein D3, a trimethyl demethylase for H3K27me3 in carcinoma cell lines [35]. It can also recruit DNA methyltransferase (DNMT)3A, as shown in gall bladder cells, leading to the decreased expression of CDH1 coding for E-cadherin [36]. However, YAP activation also induces and recruits ten-eleven translocation (TET)1, a DNA demethylation enzyme, via an interaction with TEAD; it also promotes histone H3K27 acetylation, hence increasing chromatin accessibility at YAP target genes, as shown in the liver [37].

In a previous analysis of the literature, we had concluded that the impact of AJC shuttling proteins on the genome was different between the absence and the presence of apical polarity [13]. One possible explanation, as illustrated above with Ybx3 and YAP, was that nonshuttling TFs released upon apical polarity disruption would alter chromatin organization at target genes, hence modifying the local epigenetic context for the action of shuttling AJC proteins [13]. Another nonexclusive possibility to consider is that altered homeostasis associated with prolonged apical polarity disruption is due to changes in HOCO.

### Elements related to AJC influence HOCO toward chromatin opening

Chromatin de-condensation (or relaxation) may occur through histone acetylation or phosphorylation that decreases positive charges on histones, hence potentially disrupting electrostatic interactions between histones and the DNA and changing the map of epigenetic compartments. YAP is found at enhancers, where it interacts with TEAD4 and H3K4me1, contributing to histone acetylation by p300/CREB-binding protein (CBP). The propagation of H3K27 acetylation to neighboring enhancers creates super-enhancers (or clustered enhancers with open chromatin). These enhancers include a large quantity of macromolecules (e.g. noncoding RNAs and TFs) to rapidly promote the transcription of genes necessary for homeostasis, as is the case during inflammatory response [38]. However, macromolecules may also reduce the expression of TJ components, as shown in the colon [39]. Thus, chromatin opening that is typically associated with the promotion of transcription still permits the down-regulation of certain genes depending on the local environment of macromolecules.

The disruption of apical polarity also alters the interaction of Par6–protein kinase C (PKC)–Cdc42, leading to the sustained activation of mitogen-activated protein kinase (MAPK). The Ras-MAPK pathway promotes mitogen- and stress-activated kinases and c-Jun N-terminal kinase that are involved in the phosphorylation of histone 3 (H3) at serines 10 and 28 in the regulatory regions of genes. It happens notably in immediate early genes (e.g. c-JUN, -FOS, and -MYC) involved in differentiation control, in turn promoting chromatin remodeling and transcription [40–42].

We recently reported that loss of apical polarity is characterized by a strong decrease in RMP in breast luminal cells [4]. This finding is in line with reports on depolarization being involved in dedifferentiation and tumorigenesis through the regulation of transcriptional networks [43]. Depolarization has been associated with increased calcium entry via voltage-sensitive channels [26]. The concentration of Ca^2+^ regulates H3 acetylation and phosphorylation [44,45], notably via protein kinase C β activation. Phosphorylation on H3T6 prevents the demethylation of H3 at lysine 4 by histone demethylase LSD1, hence favoring transcription [46]. Moreover, Ca^2+^ acts as a biochemical regulator for molecular binding [47], as exemplified by the activation of TFs (like CREB1), leading to the recruitment of chromatin remodeling complexes [48]. Interestingly, the impact of the RMP on differentiation and proliferation control is linked not only to a cell’s own voltage potential but also to the voltage potential of neighboring cells through electrical synapses represented by the gap junctions [49]. Hence, the loss of apical polarity that seems to systematically involve gap junction disruption in the breast luminal cells will influence voltage potential gradients, possibly leading to downstream effects with different gene alteration profiles. This possibility would represent a source of phenotypic heterogeneity in the affected tissue with potential consequences on tissue homeostasis.

### Prolonged chromatin opening is a source of instability

Most of the chromatin alterations reported upon activation of biochemical, bioelectrical, and mechanotransduction (e.g. YAP) pathways by apical polarity loss are toward chromatin opening or relaxation, a phenomenon that was thought early on to have implications in breast cancer [50]. It is confirmed in light of dysfunctional ‘enhancer reprogramming’ that causes plasticity and extended abnormal gene expression [51]. Chromatin relaxation might be reflected by the increased area of DAPI staining that we observe in apical polarity-disrupted cells primed to enter the cell cycle, although still negative for the Ki67 cycling marker [4].

Disruption of apical polarity may not be associated with changes in HOCO sufficient to perturb fundamental differentiation-built nuclear organization. We are making this statement because of the reversibility of induced loss of apical polarity [52], and the fact that long-range chromatin contacts and inter-TAD contacts associated with replication domains are unchanged when comparing quiescent and cycling cells [53,54]. However, a relaxed genome is a destabilized HOCO [50], the extreme case being that of totipotent cells displaying weakened enhancer–promoter interactions and TAD insulation [21]. Whether changes of such nature occur with prolonged loss of apical polarity remains to be determined.

Even without obvious phenotypic changes (like cells actively cycling), the opening of chromatin associated with apical polarity loss is likely to contribute to the progressive chromatin changes necessary for oncogenesis (i.e. activation of oncogenes and inactivation of tumor suppressors). At least part of the open chromatin linked to apical polarity disruption results from the targeting of genes by specific TFs. Some of these are considered oncogenic factors, as is the case for YAP, whose contribution through interaction with TEAD and promotion of H3K27 acetylation has been definitely associated with tumorigenesis [37]. YAP activation also induced the expression of TET1, leading to DNA demethylation after interaction with TEAD and chromatin opening [37]. Yet, DNA with low methylation levels is prone to increased chromosomal instability [55]. Therefore, correcting disturbed homeostasis should start early, when apical polarity appears to be altered.

## Part 2: networking for polarity recovery

Apical polarity is rapidly disrupted by exposure to increased matrix stiffness, and inflammatory and oxidative molecules, all associated with breast cancer risk [2,3,22,56]. Canceling pro-oxidative leptin levels [52] or the expression of an oncogene [57] that drove apical polarity loss in experimental settings re-establishes polarity. In life, such exposures are usually chronic, with sustained polarity disruption and time to establish permanent epigenetic drift, thereby creating a favorable soil for disorders. There are different degrees of apical polarity loss observed both in 3D cell culture models and in normal and preinvasive breast tissues [2,57]; therefore, restoring homeostasis might require different approaches. In the next sections, we discuss potential directions for recovering homeostasis in connection with apical polarity.

### Incremental alterations in apical polarity are associated with escalating phenotypic changes

The loss of polarity involves TJ disruption followed by misorientation of the spindle pole, with epithelial stratification and filling of the lumen [2,57]. On breast biopsy sections, luminal stratification correlates with the absence of Cx43 [2].

The disruption of TJ may occur through PI3K activation with Cx43 still expressed but relocated away from lateroapical sites, as shown in 3D cell culture [2,3]. Such relocation could be linked to Cx43 phosphorylation on serines S255 and S262, shown to be inhibitory for gap junction intercellular communication (GJIC) in models other than the breast [58,59]. Indeed, downstream of PI3K, PKCζ phosphorylates Cx43 on S262 [60], and the TJ disruption-induced MAPK pathway acts on S255 and S262 [61,62]. In such conditions, cells seem only primed to enter the cell cycle, since treatment with insulin growth factor 1 is necessary to induce Ki67 expression [2,3]. Importantly, downstream of PI3K, adding to other impacts of apical polarity loss on chromatin described earlier, Akt activation may promote chromatin accessibility and opening through the phosphorylation of epigenetic enzymes [63]. Examples include DNMT1, which disrupts the DNMT1/UHRF1/PCNA complex and reduces 5mC methylation globally and locally [64], EZH2, which decreases H3K27me3 [65], lysine demethylase 5A, which increases H3K4me3 [66], and p300/CPB, which increases H3 acetylation [67] (Table 1).

**Table 1:** Epigenetic modifications mentioned in the text. Writers are enzymes adding the epigenetic modification; erasers are enzymes removing the modification. Details on the exact names of the epigenetic enzymes involved are not included since there are sometimes several types for one category, with variations in their structure, number of epigenetic modifications added or removed, cell type, and differentiation state.

Modification	Writers	Erasers	Effect of the modification if ‘on’
DNA methylation	DNMT1 (maintenance) and DNMT3A (*de novo*)	TET1 and 2	Gene silencing (in promoters)
H3K4ac	HATs	HDACs	Gene activation
H3K4me1	KMTs	KDMs	Gene activation
H3K4me3	KMTs	KDMs	Gene activation
H3K9me3	G9a	KDMs	Gene silencing (strong)
H3K27ac	p300, CBP, and P/CAF	HDACs	Gene activation
H3K27me3	EZH1/2	JMJD3	Gene silencing (weak)
H3S10P	MSK	Phosphatases	Gene activation
H3S28P	MSK	Phosphatases	Gene activation
H3T6P	PRKCB	Phosphatases	Gene activation
H4K20me3	KMTs	KDMs	Gene silencing

ac, acetylation. CBP, CREB-binding protein. DNMT, DNA methyltransferase. EZH1/2, enhancer of zeste homolog 1/2. H, histone. HAT, histone acetyltransferase. HDAC, histone deacetylase. JMJD3, Jumonji domain-containing protein D3. K, lysine. KDM, lysine demethylase. KMT, lysine methyltransferase. me, methylation. MSK, mitogen- and stress-activated protein kinase. P/CAF, P300/CBP-associated factor. P, phosphorylation. PRKCB, protein kinase C β. S, serine. T, tyrosine. TET, ten-eleven translocation.

A higher level of destabilization of the breast epithelium occurs with the silencing of Cx43 [68] that not only alters apical polarity but also promotes cell proliferation, invasive capacity, and epithelial–mesenchymal transition (EMT). The loss of Cx43 expression induces the up-regulation of miR-183-5p and miR-492 [69], and miR-182-3p [70] involved in EMT. This stronger alteration of apical polarity loss seems associated with a more severe change of HOCO. Cells only primed to enter the cell cycle and that maintain Cx43 expression, although GJIC is disrupted, display a more relaxed genome [4]; but, cells primed to undergo EMT (upon loss of Cx43) display a decrease in chromatin bulk circularity [2] that is a characteristic of cells going toward tumorigenesis [71].

### Re-expression of Cx43 restores full polarity

In MCF10A mammary epithelial cells lacking Cx43 and apical polarity, *de novo* Cx43 expression restores GJIC and the polarity axis, with lateroapically localized TJs [2]. Since there are areas of the normal breast luminal epithelium lacking Cx43 expression [2], it might be necessary to target its gene to recover apical polarity. The absence of Cx43 appears to be epigenetically driven in light of successful chemical induction of Cx43 expression in numerous cancer cells, as well as in non-neoplastic human cells. This was shown with HDAC inhibitors 4-phenylbutyrate, suberoylanilide hydroxamic acid, or trichostatin A (TSA) in neural progenitor cells [72], peritoneal mesothelial cells [73,74], and prostate epithelial cells [75]. Cell-type independent TFs, Sp1 and AP-1 also promote Cx43 expression, but they require an open chromatin (e.g. via TSA treatment) [75]. However, the treatments described here would increase chromatin opening within the already relaxed chromatin context of apical polarity loss, potentially promoting instability.

So far, there is no report of a direct effect of Cx43 on chromatin. Therefore, we propose that the combination of GJIC and Cx43 interaction with partners capable of influencing gene transcription establishes HOCO associated with full polarity in the differentiated breast epithelium. If this assumption is correct, the re-establishment of lateroapical GAP junctions might be the trigger to normal homeostasis.

### Re-establishment of homeostasis through an action on chromatin

Maintenance of cell homeostasis is best performed in a stable nuclear environment with low energy consumption. Compaction of chromatin, a characteristic of normal differentiation, preserves energy [38].

**Figure 2:**
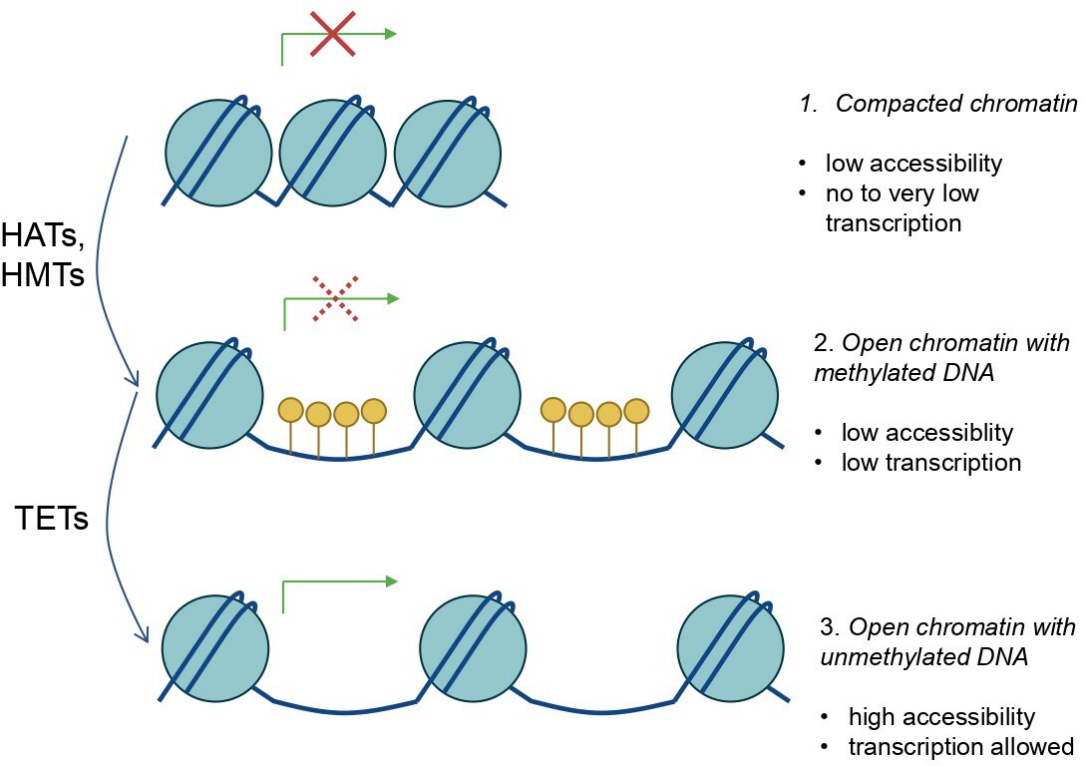
Three levels of local chromatin structure for transcription that may be influenced upon the disruption of apical polarity. There are several levels of gene transcription control through epigenetic modifications: (**1**) the chromatin is compacted through histone modifications, bringing DNA and nucleosomes close together and there is very low accessibility to DNA ; (**2**) the activation of histone acetyltransferases (HATs) and/or histone methyltransferases (HMTs) relaxes chromatin and prepares gene promoters for the binding of the transcription machinery, but the presence of methylation on the DNA leads to the recruitment of DNA binding proteins that prevent DNA accessibility and interaction with RNA polymerase II, lowering the possibility for transcription; (**3**) ten-eleven translocation (TET) demethylases remove methyl groups from the DNA on an open chromatin, giving full access to the gene; transcription is possible depending on the presence of the necessary TFs. Please note that only the type of modifiers discussed in the text of the article are listed*; *TFs, chromatin remodeling complexes and transcription complexes are not indicated on the cartoon.

Treating breast epithelial cells with the DNA demethylating agent 5-aza-2′ deoxycytidine prevents the formation of the polarity axis [76], and loss of DNMT1 in the lung endoderm prevents epithelial polarity [77], making DNA more accessible for transcription (Figure 2). If we attempt to make DNA less accessible, we could target TET DNA demethylation enzymes; inhibitors exist, but the effect on gene transcription has barely been studied [78]. Re-methylation of the DNA may not be sufficient to revert chromatin relaxation induced following apical polarity disruption, as it reduces access to gene promoters but does not recondense the genome.

Although EZH2 participates in chromatin condensation through H3K27 methylation, its absence helps maintain a polarized epithelial phenotype through expression of a Wnt signaling suppressor (SFRP1) and of Pten in a mammary organoid system [79]. This observation confirms the involvement of EZH2 in the epigenetic drift associated with cancer onset. EZH2 inhibitors are in clinical trials [80]; their effect on decondensation appears limited to H3K27me3-marked gene promoters, as shown in the context of leukemia [81]. Thus, EZH2 inhibitors might be of interest in combination with therapies that recondense phosphorylated or acetylated chromatin.

Targeting chromatin relaxation typical of apical polarity loss through an action on histone phosphorylation and acetylation might be achieved by acting on MAPK or directly on the epigenetic enzyme p300/CBP. There are inhibitors for both [82,83]. Whether such treatment would be sufficient to restore apical polarity, depending on the presence or absence of Cx43, remains to be tested. In contrast, the establishment of apical polarity would reduce Ca^2+^ concentration, hence possibly stopping histone phosphorylation and acetylation.

### Promoting differentiation to restore homeostasis

Differentiation inducers, like all-trans retinoic acid (ATRA), might be interesting. This nutrient has a direct impact on gene expression via the nuclear concentration of retinoic acid receptors (RARs). It was recently reported to rewire chromatin topology, notably accessibility and looping, in zebrafish [84]. It is effective to revert the early transformation stage in the MCF10A breast cancer progression series [85]. In particular, it can stimulate, in non-neoplastic MCF10A cells, the nuclear concentration of interferon regulatory factor-1, a tumor suppressor involved in the control of cell proliferation and immune responses [86]. ATRA also triggers RAR-TET2-activated miR-200c suppression of PKCζ expression, which promotes epithelial differentiation in the mammary stem cell pool [87].

Remarkably, ATRA is considered an agonist of Cx43. In models other than the breast, it enhances GJIC by increasing the expression of Cx43 through direct transcriptional activation of its gene, GJA1. Although no retinoic acid response element was found in the gene, Sp1 and Sp3 were bound to the transcription start site [88]. The positive effect of ATRA on Cx43 expression and GJIC was also reported with lens epithelial cells [89]. ATRA-induced GJIC is accompanied by a reduction in Cx43 phosphorylation [90,91]. Hence, ATRA might be of interest whether CX43 is present or not in the mammary epithelium deprived of apical polarity.

The use of ATRA with the EZH2 inhibitor GSH343 suppresses synovial sarcoma cell proliferation [92], which suggests that combining biochemical and confined epigenetic actions might be interesting to investigate. However, many examples of combinations of inhibitors of pathways of interest for homeostasis linked to apical polarity involve cancer cells. For instance, combining MAPK and Pi3K inhibitors *in vitro* and in mice impairs tumor growth [93–96]. The concept for such a combination is that targeting both pathways simultaneously prevents compensatory feedback [97]. It seems that none of these studies investigated an impact on chromatin. Combining inhibitors of MAPK–ERK, EZH2, and BRD4 (a transcription regulator) led to tumor growth reduction and diminished H3K27me3 and H3K27ac [98]; it also decreased acetylation that is expected from MAPK inhibition, suggesting chromatin compaction. Whether such combinations might be feasible to re-establish apical polarity remains to be investigated.

## Conclusion

In ‘homeostasis in the breast: it takes a village’, Rizki and Bissell compare homeostasis to a collection of members (different cell types) working in harmony [99]. Here, we have looked specifically at the homeostasis of the breast luminal epithelium, a tissue in which the basoapical polarity axis is a protective barrier against cancer onset. The recent demonstrations that Cx43-mediated GJIC controls apical polarity [2] and the RMP [4] reveal Cx43 as a cornerstone of the homeostasis of polarized luminal cells. Yet, homeostasis requires the tight control of gene transcription. Although Cx43 does not appear to act directly on genes, it is through networks of protein–protein interactions, signaling pathways, and elements involved in chromatin remodeling that homeostasis is maintained. Pieces of the puzzle are missing, especially for HOCO or genome organization, itself considered part of a ‘network of networks’ [100]. Pioneering work on breast cancer prevention had envisioned ATRA as a possible therapeutic candidate [101,102]; this prospect seems to have faded, although a synthetic retinoid, fenretinide, has shown promises preclinically and clinically [103]. The connection between ATRA and Cx43 might reignite interest within the network of scientists and oncologists empowered by the shared goal of moving forward the primary prevention of cancer. In light of the change of membrane potential in cancer cells, bioelectrical properties of cells are emerging as potential targets to treat cancer using ion channel modulators [104]. Whether acting on these channels in conjunction with Cx43-mediated GJIC might help restore full polarity and homeostasis is a potentially interesting direction of research.

**Perspectives**

- Apical polarity controls epithelial homeostasis, it is influenced by several breast cancer risk factors, and its loss is a prerequisite to breast cancer development. Understanding the mechanisms of polarity-mediated homeostasis could lead to therapeutic innovations in breast cancer prevention.
- Through gap junctions and cell–cell adhesion complexes, apical polarity controls signaling and bioelectrical pathways, as well as transcription factors that all have an impact on chromatin organization, forming a complex regulatory network. There are different degrees of apical polarity disruption with different degrees of homeostatic disturbance.
- It seems important to investigate how various risk factors affect apical polarity in order to optimize the development of means to reestablish full differentiation and homeostasis, possibly by combining different approaches to sustainably correct epigenetic alterations.

## Abbreviations

AJC: apical junctional complex
ATRA: all-trans retinoic acid
CBP: CREB-binding protein
CDK4: cyclin-dependent kinase 4
Cx43: Connexin 43
3D: three-dimensional
DNMT: DNA methyltransferase
EMT: epithelial-mesenchymal transition
EZH2: enhancer of zeste homolog 2
GJIC: gap junction intercellular communication
HDAC: histone deacetylase
HOCO: higher order of chromatin organization
MAPK: mitogen-activated protein kinase
PCNA: proliferating cell nuclear antigen
PI3K: phosphoinositide 3-kinase
RAR: retinoic acid receptor
RMP: resting membrane potential
TAD: topologically associating domains
TET: ten-eleven translocation
TF: transcription factor
TJ: tight junction
TSA: trichostatin A
YAP: yes-associated protein
Ybx3: Y-box binding protein 3
ZO: zonula occludens
